# Milk and Dairy Products and Their Nutritional Contribution to the Average Polish Diet

**DOI:** 10.3390/nu11081771

**Published:** 2019-08-01

**Authors:** Hanna Górska-Warsewicz, Krystyna Rejman, Wacław Laskowski, Maksymilian Czeczotko

**Affiliations:** Department of Organization and Consumption Economics, Faculty of Human Nutrition and Consumer Sciences, Warsaw University of Life Sciences, 02-787 Warsaw, Poland

**Keywords:** milk, dairy products, energy intake, nutrient intake, food sources

## Abstract

The main aim of this study was to identify the dairy sources of energy and 44 nutrients in the average Polish diet. Our research included: carbohydrates, protein, total fat, saturated fatty acids (SFA), monounsaturated fatty acids (MUFA), polyunsaturated fatty acids (PUFA), cholesterol, 18 amino acids, 9 minerals, and 10 vitamins. The analysis was conducted based on the data from the 2016 Household Budget Survey, a representative sample of the Polish population (i.e., 36,886 households). The category of milk and dairy products was divided into three main groups (i.e., milk, cheeses, and yoghurts, milk drinks and other dairy products) and seven sub-groups (i.e., whole milk, reduced fat milk, condensed and powdered milk, ripened and melted cheese, cottage cheese, yoghurts, milk drinks and other dairy products). Milk and dairy products provided 9.1% of the total energy supply. A high share (above 20%) in the supply of nutrients was noted in the case of calcium (54.7%), riboflavin (28.1%), vitamin B12 (26.1%), and phosphorus (24.6%). Supply at the level of 10–20% was observed for protein, SFA, zinc, total fat, cholesterol, potassium, magnesium, and vitamin A. Of the amino acids, the share above 20% from dairy category was recorded in the case of 6 amino acids (proline, tyrosine, serine, lysine, valine, and leucine) and at the level of 10–20% for 10 amino acids (isoleucine, histidine, threonine, tryptophan, phenylalanine, methionine, glutamic acid, aspartic acid, alanine, and arginine).

## 1. Introduction

Milk has been known as nature’s most complete food [1] for millennia, playing currently an important role in the diet of over 6 billion people in the world [2,3,4]. Bovine milk predominates (83%) in global milk production, which has increased over three decades by ca. 60%, from 522 million tons to 828 million tons in 1987–2017 [5], in order to meet the growing demand for dairy products. According to Organization for Economic Cooperation and Development (OECD) and Food and Agriculture Organization (FAO) projections, the milk and dairy product consumption level per capita (in milk equivalent) in the coming years should remain very high in western countries in the regions of Europe and North America, while a significant increase is shown in North African countries, the Middle East and an exceptional rise in Asia zone countries and the East. A considerable rise in demand in central and east European Union countries (new member states of the EU), in the United States and Russia is also predicted [6].

Milk and dairy products are nutrient-dense foods, supplying energy and high-quality protein with a range of essential micronutrients (especially calcium, magnesium, potassium, zinc, and phosphorus) in an easily absorbed form [7,8,9,10,11,12,13]. Milk minerals are crucial for human health and development as well as in dairy processes as cheese-making and for all traits involving salt-protein interactions [14]. They play a key role in healthy human nutrition and development throughout life, but especially in childhood [7]. Dairy products are rich in nutrients that are essential for good bone health, including calcium, protein, vitamin D, potassium, and phosphorus [8]. Adequate calcium intake influences skeletal calcium retention during growth and thus affects peak bone mass achieved in early adulthood [15,16]. The high levels of calcium play an important role in the development, strength, and density of bones for children and in the prevention of bone loss and osteoporotic fractures in elderly people [1,8,16]. Studies show that frequent consumption of dairy foods and milk should be recommended in order to prevent periodontal disease [17,18,19,20,21]. Calcium also has been shown to be beneficial in reducing cholesterol absorption, and in controlling body weight and blood pressure [1].

There is no such unequivocal position regarding the effect of milk and dairy fat on human health. Currently controversy has emerged about the benefits compared with harms of dairy fat, including concerns over long-term effects [22]. The traditional diet-heart paradigm held that consumption of fat, and saturated fat in particular, raised levels of total and low-density lipoprotein (LDL) cholesterol leading to coronary heart disease. Following this knowledge dietary guidelines in many countries and of international authorities recommend consumption of low-fat dairy foods [7]. However recent studies show that milk and dairy products with high fat contents do not increase total and LDL cholesterol levels [23,24], and whole milk significantly increases high-density lipoprotein HDL cholesterol concentrations compared to skimmed milk [24]. Most meta-analyses report no or weak inverse association between dairy intake, including butter, with cardiovascular disease and related intermediate outcomes [25,26,27]. However, some original studies indicate that saturated and trans fatty acids from dairy products were inversely associated with adiposity, diabetes and inflammatory outcomes, suggesting that dairy fat intake may have a beneficial effect on cardiovascular health [22,26,28,29]. Long-term cheese consumption is not associated with an increased risk of all-cause mortality [30] and even high consumption of dairy products, especially yogurt and cheese, may reduce the risk of overall and cardiovascular disease (CVD) mortality [31]. The relation between dairy product intake and the risk of ischemic heart disease remains controversial [28]. There is some suggestion that dairy consumption is inversely associated with stroke incidence [22,27].

As regards to type 2 diabetes, findings are inconsistent. Studies showed that higher intake of yogurt is associated with risk reduction of this disease [27,32,33], also a significant inverse association between intakes of dairy products, low-fat dairy products, and cheese and risk of type 2 diabetes was observed on the base of meta-analysis of 17 cohort studies [34]. On the other hand, findings indicated that consumption of total dairy are not beneficial in risk reduction of type 2 diabetes [32] and the associations varied both by dairy product and type and by baseline glycaemic status of the population [35].

Total dairy product intake has no significant impact on increased overall cancer mortality risk (results of meta-analysis study) [31,36] nor does the intake of individual dairy products [31], however a relationship existed between increase of whole milk intake and increase of prostate cancer mortality risk [36]. The Third Expert Report “Diet, Nutrition, Physical Activity and Cancer: a Global Perspective” [37] in a certain sense reflects this finding, showing that currently there is only limited evidence that dairy products (total dairy, milk, cheese and yogurt) and diets high in calcium increase risk of prostate cancer. According to these report findings, there is strong evidence that consuming dairy products (total dairy, milk, cheese and dietary calcium) decreases risk of colorectal cancer. This inverse association is largely attributed to high calcium content in milk and its products. In addition to calcium, lactic acid-producing bacteria may also protect against colorectal cancer, while the casein and lactose may increase calcium bioavailability. Other nutrients or bioactive constituents in dairy products, such as lactoferrin, vitamin D or the short-chain fatty acid butyrate may also impart their beneficial functions against colorectal cancer. 

Aside from nutritional values of milk and dairy products their biologically active compounds (bioactive peptides, probiotic bacteria, antioxidants, vitamins, specific proteins, oligosaccharides, organic acids, highly absorbable calcium, conjugated linoleic acid and others) have crucial impacts on human functioning and health [1,25,38,39,40,41,42,43,44].

In terms of the importance of milk and dairy products, changes in food choice determinants and consumption patterns, the analysis of energy and nutrient sources in a given population is crucial to assure the adequate nutritional quality of diets. The aim of this study was to determine the role of milk and various types of dairy products as sources of energy and 44 nutrients in the average Polish diet based on data from the 2016 Household Budget Survey conducted by the Central Statistical Office on the representative sample of the Polish population.

## 2. Methods

### 2.1. Study Overview

Sources of energy and nutrients from milk and dairy products were analyzed. The 44 nutrients examined in this study included: carbohydrates, protein, 18 amino acids (leucine, isoleucine, valine, lysine, histidine, threonine, tryptophan, phenylalanine, methionine, cysteine, tyrosine, arginine, alanine, aspartic acid, glutamic acid, glycine, proline, and serine), total fat, fatty acids (saturated fatty acids, SFA; monounsaturated fatty acids, MUFA; polyunsaturated fatty acids, PUFA), cholesterol, 9 minerals (calcium, phosphorus, sodium, potassium, magnesium, iron, zinc, copper, iodine), and 10 vitamins (thiamin, riboflavin, niacin, vitamin B6, folate, vitamin B12, vitamin A, vitamin D, vitamin E, and vitamin C). 

The research process is presented in Table 1 and described in sub-chapters 2.2, 2.3 and 2.4.

### 2.2. Sample Selection Method 

The Household Budget Survey (HBS) is a representative method of data collection in Poland organized and conducted by the Central Statistical Office, Social Surveys and Living Conditions Statistics Department. This survey is carried out annually on a sample of 36–39 thousand households. In this study, we used the 2016 data for 36,886 households (total number of persons: n = 99,230) participating in the HBS [45]. Every year the research sample is selected randomly on the basis of a two-stage randomization system covering areas survey points, flats and households. This allows generalizing the received results for all households in Poland. Since 1993, the HBS has been conducted using a total monthly rotation [45,46,47,48].

In each household participating in the survey, the data of revenues, and consumption expressed in terms of quantity (in grams, kilograms, liters) and value (in Polish zloty PLN) are recorded in special budget book (“Household Budget Diary”) for one month. Based on the HBS information on the number of persons in each household and the number of days of in-home nutrition, we calculated the consumption per one person per month for each household. We have received the amount of consumption expressed in grams, kilograms or liters per one person per month and day for each of 91 sub-groups.

Detailed information related to the sampling system, sample selection, research tools, and method of collecting information is provided in our previous studies [49,50,51].

### 2.3. Food Grouping 

The HBS database contains the quantities of purchased and consumed food products from 91 food sub-groups (in grams, kilograms, liters) per month in each of 36,886 households. The classification of food products was developed based on a literature review [45,52,53,54,55], the specificity of products available on the Polish market [45,46] and was published in our earlier studies [49,50,51]. For the purpose of this study, we have included the dairy category “milk and dairy products” divided into three main groups and seven sub-groups (Table 2).

### 2.4. Statistical Analysis

The next step was to convert the quantitative consumption of milk and dairy products (in grams, kilograms and liters) into the supply of energy and 44 nutrients to the diet in each household. This was the basis for calculating the average energy and nutrient supply for all households taking part in the HBS. For these calculations, we used current version of nutritional value tables, Nutritive Value Tables for Foods and Meals” (4th ed.) [56] as well as the R program (v 3.0.2) (Copyright (C) 2018, The R Foundation for Statistical Computing, Vienna, Austria), system and environment for statistical computation [57,58,59]. The weights of corrections were implemented to improve the representativeness of the results. It was possible due to a programming language with conditionals, loops, user defined recursive functions as well as a coherent, integrated tool collection for data analysis included in the R program [57,59]. This allowed us to consider the results as being representative for the Polish population [47,48]. We presented the results as a percentage supply of energy and 44 nutrients from the category "milk and dairy products". The details about this conversion are also included in our previous studies [49,50,51].

To analyze the impact of socio-demographic and economic factors on the level and structure of energy and nutrients supply from dairy category in the average diet, a cluster analysis as an exploratory tool [60,61,62] was applied. We included the level of the consumption of main dairy product groups to divide the sample population into clusters. In our calculations, the Neural Networks module in the Statistica 13.3 program (Copyright 1984-2017, TIBCO Software Inc., Palo Alto, CA, USA) and Kohonen Neural Network was applied [63]. The division into three clusters is characterized by an averaged correlation measure (correlation ratio) at a level of almost 0.5.

The identification of clusters was based on the following socio-demographic and economic features: age, sex, socio-economic affiliation/type of the households, education, income measured according to the quintile groups, assessment of the household’s financial situation, assessment of nutrition, number of people in the household, number of inhabitants in the village, land use, family life phase, degree of urbanization, region, and study month. For each feature, the correlation table was created together with a measure of Cramer’s correlation. 

## 3. Results

Dairy sources of energy (Table 3) protein and amino acids (Table 4), carbohydrates, total fat, SFA, MUFA, PUFA and cholesterol (Table 5), minerals (Table 6), and vitamins (Table 7) are presented in the following sub-chapters.

### 3.1. Milk and Dairy Products as Sources of Energy

Energy contribution from milk and dairy products amounted to 9.1% (Table 3), which meant energy supply from dairy products at 205 kcal in the average Polish diet providing 2261 kcal. The largest share in the energy supply was characterized by cheeses (including ripened, melted and cottage cheeses), followed by milk (in particular, whole fat milk).

### 3.2. Milk and Dairy Products as Sources of Protein, Amino Acids, Carbohydrates, Total Fats, Fatty Acids, and Cholesterol

Milk and dairy products provided 18.1% of the daily total protein supply (Table 4). The supply of branched-chain amino acids (leucine, isoleucine and valine) from milk and its products developed at the level of 19–21%. The remaining essential amino acids were supplied by milk and dairy products in an amount from 18.4% for methionine to 21.5% for lysine. In terms of the contribution of non-essential amino acids, differences in the supply of milk and dairy products from 7.8% (glycine) to 23.6% (tyrosine) were observed. The lowest supply of milk and dairy products was recorded for glycine (7.8% of total supply) and cysteine (8.5%), the largest for tyrosine (23.6%) and proline (24.8%).

The structure of supply of particular amino acids indicates the significant position of cheeses. The cheeses were responsible for almost 9% of total protein supply. Considering branched-chain amino acids and other essential amino acids, the proportion of cheeses in the supply ranged from 8.6% (threonine) to 10.1% (valine). The second position was taken by milk, and the third—by milk drinks and other dairy products.

Dairy sources of carbohydrates, total fat, fatty acids (SFA, MUFA, PUFA), and cholesterol are shown in Table 5. Milk and dairy products provided 11.3% of the total supply of total fat with a focus on SFA (18.4%). The share of milk and dairy products in the supply of MUFA and PUFA was at 8.8% and 2.1% respectively. When analyzing product groups, the dominant share of cheeses in average supply of total fat (6%) and SFA (9.9%) should be indicated. The share of ripened and melted cheeses was at the level of 4.6% and 7.5%, respectively. In the supply of cholesterol, the share of milk and dairy products was 11.6%, of which almost half were cheeses (5.7%). The category of dairy products was responsible for the supply of carbohydrates in 5%. The most important ones were yoghurts, milk drinks and other dairy products (2.4%), and milk (2.3%).

### 3.3. Milk and Dairy Products as Sources of Minerals and Vitamins

Milk and dairy products are an important source of calcium, accounting for almost 55% of the supply of this mineral (Table 6). A large share was characterized by two product sub-groups: milk and cheese (each of these sub-groups provided over 20% of the total supply of this mineral). The supply of phosphorus from the category of milk and its products amounted to almost 25%, of which cheese accounted for more than 10%. The share of milk in the supply of phosphorus was at the level of 9.4%. In the case of potassium, magnesium and zinc, the share of milk and its products in the average daily intake was in the range of 10–14%. In the case of potassium and magnesium, milk predominated with a share of 6.8% in the case of potassium and 5.6% for magnesium, respectively. For the supply of zinc from milk and dairy products, cheese was the most important source of this mineral (6.7% supply of this mineral in the average Polish diet). The remaining minerals were supplied by milk and its products at the level of 7–9%.

The largest share of milk and its products in the supply of vitamins was recorded in the case of riboflavin and vitamin B12 (Table 7). In the structure of supply, the first place was for milk, providing 13.5% of the total supply of riboflavin and 10.3% of vitamin B12. The supply of vitamin A from the dairy category was at the level of 11%, of which 5.2% constituted cheese, and 4.4%—milk. The remaining vitamins were delivered by milk and its products at a level of less than 10%.

### 3.4. Summary

**Figure 1:**
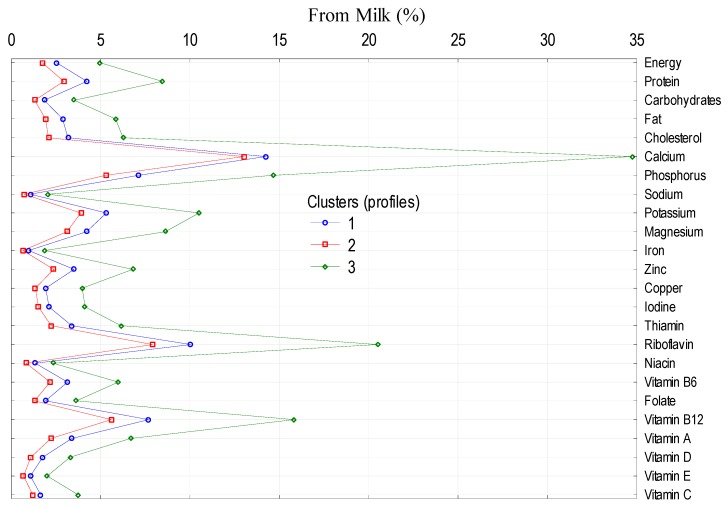
Cluster analysis: supply of energy and nutrients to the average Polish diet.

**Figure 2:**
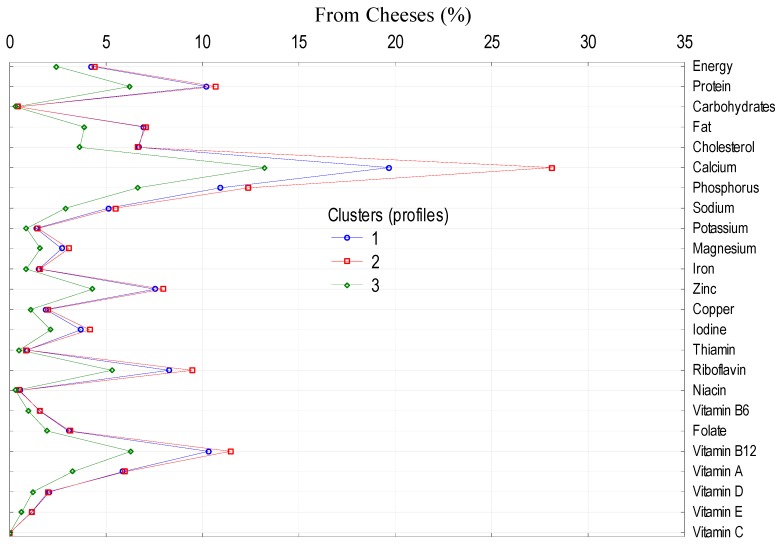
Cluster analysis: supply of energy and nutrients from cheeses to the average Polish diet.

**Figure 3:**
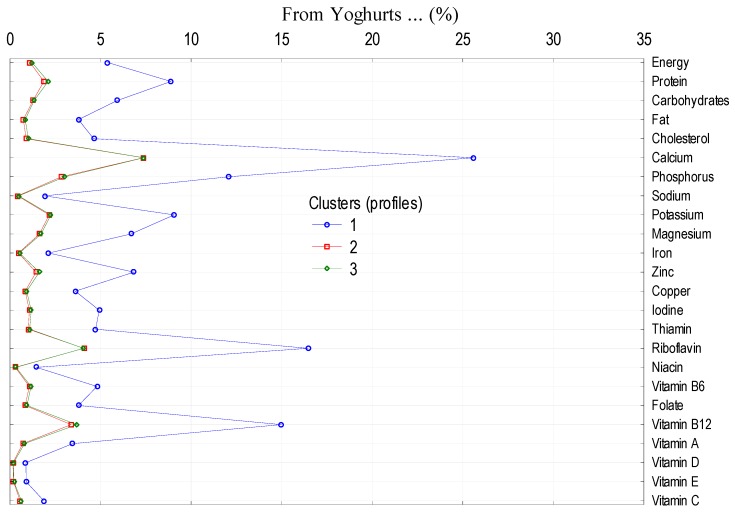
Cluster analysis: supply of energy and nutrients from yoghurts, milk drinks and other dairy products to the average Polish diet.

To analyze the impact of socio-demographic and economic features of the households on the level and structure of energy and nutrient supply from dairy category in the average Polish diet, main dairy product groups (milk, cheeses as well as yoghurts, milk drinks, and other dairy products) were selected. The largest impact was observed in the case of the following factors: size of the village, socio-economic affiliation of the household, education, and land use (Table 8). Clusters differ in the consumption of milk, cheeses, as well as yoghurts, milk drinks and other dairy products (Table 9), which differentiates the supply of energy and nutrients from these products to the average diet (Figure 1, Figure 2 and Figure 3).

## 4. Discussion

Milk and dairy products are an important food category in the structure of the Polish diet. The aim of this study was to determine the importance of this product category in the supply of energy, macronutrients (including amino acids), minerals and vitamins. Particular attention was paid to product groups (i.e., milk, cheeses as well as yoghurts, and milk drinks) and sub-groups (e.g., whole milk, reduced fat milk, condensed and powdered milk, ripened and melted cheeses, cottage cheeses, yoghurts as well as milk drinks and other dairy products). The obtained results were compared with the results described in the scientific literature referring to other populations, including the American [53,64], Spanish [65,66,67,68], Dutch [69,70], New Zealand [71] and Australian [72,73,74] population.

In the structure of energy supply to the average Polish diet, the share of milk and its products was at the level of 9.1%, providing 205 kcal from the total energy value of the Polish diet. The largest share of dairy products in supplying energy was cheese (3.7%) and milk (3.2%). In the average American diet, milk and dairy products provided 10.6% of the total energy supply. The share of cheeses amounted to 6.8%, milk—3.8%, and dairy desserts—2.2% [53]. Detailed data from 2007–2010 indicate the supply of energy from dairy products in the following order: cheese (ripened cheese: 2.6%, macaroni and cheese: 0.8%, cottage/ricotta cheese: 0.4%), milk (reduced fat: 2.2%, nonfat: 1.3%, whole fat: 1.0% and low fat 1.0%), ice cream, frozen dairy desserts (1.9%), and yoghurts (0.5%) [75]. In the population of Americans over 51 years, the share of milk and dairy products in energy supply was calculated at 6.5%, including the two main product sub-groups, i.e., milk 2.8% and cheese 2.2% [64]. In the study of the population from New Zealand, similar results were obtained in comparison to the average Polish diet. The total share of dairy products in the energy supply amounted to 9.4%, of which milk provided 5% of the total energy supply, cheese—2.5% and dairy products—1.9% [71].

According to our research, milk and dairy products provided 18.1% of total protein supply in the average Polish diet, of which almost half are made of rennet, melted and cottage cheese (9.0%). In the average American diet, the share of cheeses in total protein supply was 8.5%, whole milk—6.9% [53]. Detailed research from 2007–2010 regarding the importance of dairy products in the American diet indicated a varied supply of protein from dairy products, including cheese (ripened cheese: 4.3%, macaroni and cheese: 1.0%, cottage/ricotta cheese: 0.1%), milk (reduced fat: 1.3%, nonfat: 0.6%, whole fat: 0.7% and low fat 0.5%), ice cream and frozen dairy desserts (1.0%) and yoghurts (0.7%) [75]. In the New Zealand population, the share of milk and its products in the supply of protein was 13.9%, including milk 8.8%, cheese 3.1%, and dairy products 2.0% [71]. The significance of protein supply to the average diet is a very important issue, therefore this aspect combined with energy supply is included in the nutrient density indicators used to analyze the quality of diets [76], i.e., NQI (Nutritional Quality Index of foods) [76,77,78], RRR score (ratio of recommended to restricted food score) [76], calories-for-nutrient (CFN) score [76], and naturally nutrient rich (NNR) score [76,77].

Considering the supply of protein in the diet, special attention should be paid to the supply of amino acids from the dairy category in general and for individual dairy products. In the average Polish diet, the share of milk and dairy products in the supply of branched-chain amino acids (leucine, isoleucine and valine, BCAAs) ranged from 19 to 21%. This is especially important because of the role of BCAAs in muscle protein synthesis [79,80,81].

In the average Polish diet, the share of milk and milk products in the supply of total fat was 11.3%, with SFA reaching almost 18.5%, and for MUFA—8.8%. The structure of total fat supply in general was dominated by cheeses, above all ripened and melted, while the share of milk amounted to 3.8%. When comparing the role of the dairy category, it should be emphasized that in the average American diet, the share of cheeses was 8.8% in the supply of total fats. Milk delivered 3.7% of total fat supply, and milk desserts—2.7% [53]. In the population of Americans over the age of 51, milk and dairy products provided 7.5% of the total supply of fat, and 14.1% of SFA [64]. In contrast, in the New Zealand diet, the share of milk and dairy products in the total supply of fats amounted to 12.4% with particular reference to milk—5%, cheeses—4.1% and dairy products—3.3%. SFA supply from milk and dairy products amounted to 18.6%, including 7.6% of milk, 6.3% of cheese and 4.7% of dairy products [71].

The supply of cholesterol from milk and dairy products in the average Polish diet was at the level of 11.6%, of which cheese provided 5.7% of the total daily intake of cholesterol, and milk—4.1%. Studies conducted in the American population indicate the supply of cholesterol from the dairy products category at the similar level (11.2%) [82]. A slightly higher level of supply of cholesterol from milk and dairy products was recorded in the New Zealand population (14.3%), including milk (8%), dairy products (3.5%), and cheese (2.8%) [71].

Of the analyzed minerals, special attention should be paid to calcium. In the average Polish diet, milk and dairy products provided more than half of the total supply of calcium. The role of milk and cheese is comparable, in both cases there was a share in supply at the level of about 21.5%. However, the share of yoghurts, milk drinks and other dairy products was almost 12%. Similar results were obtained for the American population, the share of milk in the supply of calcium was 22.5%, cheese—21.6%, milk desserts 3.5%, and milk drinks—2.0% [53]. Subsequent studies conducted in the United States on a sample of adults over 51 years indicate a combined share of milk and dairy products in the supply of calcium at 32.8% [64]. In the average Spanish diet, the share of milk and dairy products in the supply of calcium was at a similar level, which in Poland amounted to 53.1% [67]. A slightly higher index was obtained for the Dutch population including persons aged 7–69, the share of dairy products in the calcium supply amounted to 58% [69,70]. In the New Zealand population, the share of milk and its products in the supply of calcium amounted to 40.3%, of which the share of milk was at the level of 26.8%, cheese—7.7%, and dairy products—5.8% [71]. The analysis of calcium supply from milk and dairy products is particularly important in the context of the importance of this nutrient for good bone health [8,15,16], in reducing cholesterol absorption, controlling body weight and blood pressure [1], and preventing periodontal disease [17,18,19,20,21].

Changes in the consumption of milk and dairy products affect the supply of calcium, which, due to the importance of calcium in metabolic processes [83], may have negative health implications. Therefore, calcium (like protein) is taken into account in the indicators of nutritional density, i.e., NQI (Nutritional Quality Index of foods), RRR score (ratio of recommended to restricted food score), calories-for-nutrient (CFN) score, and naturally nutrient rich (NNR) score [76,77].

According to our research, milk and dairy products provided almost ¼ of phosphorus supply, including 10.1% of cheese, and 9.4% of milk. Similar results were achieved for the average American diet, milk provided 12.3% of phosphorus, cheese—11.3%, while milk desserts—2.0% [53]. In the average Dutch diet (data covering people aged 7–69), the share of dairy products in phosphorus supply amounted to 32% [69,70]. 

The share of milk and dairy products at the level of 10%–15% in the supply of minerals to the average Polish diet was noted for zinc (13.9%), potassium (11.9%) and magnesium (11.0%). According to Spanish study, milk and its products provided a higher percentage of zinc to the average diet (16%) [67]. Other studies indicate the supply of zinc from milk and its products at 12-15% in the Australian diet [72,73], and 13% in the average American diet [84]. In the average New Zealand diet, the share of milk and its products in the supply of zinc was 12.4%, of which milk was responsible for 7.4% of zinc, cheeses—3.3% and dairy products—1.7% [71].

The scientific literature indicates the importance of adequate supply of zinc in growth and development, immunological processes, reproductive immunity, neurological function and reproduction [74,85,86]. The importance of milk and dairy products in the zinc supply in the population is also underlined [67,74,85,86,87,88,89]. For example, in studies on the sources of zinc in the diet of Australian children from 1995 and 2007, there was a decrease in the share of dairy products in the supply of zinc from 18–24% in 1995 to 15–20% [74]. This is the reason for including zinc in the composition of indicators assessing the nutritional quality of the diet i.e., calories-for-nutrient (CFN), and naturally nutrient rich (NNR) score [76,77,78,90].

According to our research, the share of milk and dairy products in the supply of potassium is almost 12%, with a significant share of milk. In the American diet, the share of milk in potassium supply is 9.6% [53]. These considerations should be combined with the importance of potassium in regulating blood pressure [91,92], membrane transport, energy metabolism, and cell functioning [93]. 

According to our research, milk and dairy products provided over 25% of riboflavin and vitamin B12, and 11% of vitamin A to the average Polish diet. In the supply of riboflavin, the share of milk was 13.6%, while cheese and milk drinks, including yoghurts, provided riboflavin in an amount of 7–8% of the daily supply of this vitamin. In the average New Zealand diet, the share of dairy category in the riboflavin supply exceeded 30% with milk contribution at the level of 22.9%, dairy products—4.8% and cheese—2.9% [71]. Among the analyzed dairy products in the structure of vitamin B12 supply, the first place was taken by milk (10.3% of the total daily supply of this vitamin), followed by cheese (9.5%) and yoghurts, milk drinks and other dairy products (6.4%). A similar supply of dairy products in the supply of vitamin B12 was recorded in the New Zealand population (29.5%). The share of milk in the supply of this vitamin was 20.6%, cheese 4.7%, and dairy products 4.2% [71]. In the structure of vitamin A supply from the group of dairy products, the largest share concerned cheeses, in particular ripened and melted cheeses, and milk, including whole milk. In the New Zealand population, the share of milk and its products in the supply of vitamin A was at a higher level of 15.3%. From this amount, the share of milk was 6.2%, cheeses—4.9%, and dairy products—4.7% [71].

In summary, we should point to the significance of milk and dairy products in the supply of nutrients as nutrient-dense foods. This is important due to the concentration of protein and nutrients crucial for growth and health, i.e., calcium, magnesium, potassium, zinc, and phosphorus [7,8,9,10,11,12,13]. In spite of the limitations [49,51] we mentioned in previous studies, our research is an important source of information for analyzing sources of nutrients in the average Polish diet, its structure and quality. 

## 5. Conclusions

The results of our analysis showed that milk and dairy products are especially valuable food in the average Polish diet, thus confirming that they belong to foods with particularly high nutritional density. In addition, all milk and dairy products’ ingredients are well absorbed. The exception is lactose, poorly tolerated in some people, but they can choose fermented dairy products, taking advantage of the presence of probiotic bacteria, a health-promoting bioactive ingredient. 

From the three main groups of dairy products analysed, cheese and milk provide the most energy and nutrients. These main groups are significant sources of calcium as each provides about 22% of this nutrient (the total contribution of calcium from dairy products is 55%). The role of milk and dairy products in providing calcium cannot be overestimated, but its average intake in Poland is low and is around 60% of the recommended intake. The share of milk and cheese groups in the supply of phosphorus, potassium and magnesium is twice lower. In the case of vitamins, the role of milk and dairy products in the supply of riboflavin and vitamin B12 should be emphasized. Milk provides almost twice as much riboflavin as cheese. 

The share of the third group of analyzed dairy products, i.e., yoghurts, milk drinks and other dairy products in supplying nutrients to the average Polish diet was in each case lower than milk and cheese (as the consumption is lower), with the exception of carbohydrates. The contribution of this group of dairy products was 2.4%. Paradoxically, fermented milk drinks are recommended for people intolerant to lactose, but manufacturers practice adding (too much) sugar to flavored and fruit yogurt and milk drinks, especially for children. 

The results of our analysis may be helpful in developing dietary guidelines on the amount and structure of milk and dairy products’ consumption in order to achieve better health status of the population.

## Figures and Tables

**Table 1:** Research process.

Stage	Description
1.	Two-stage random selection of representative sample of households (Central Statistical Office) a) Random selection of areas survey points based on the list of statistical regions developed in Poland for National Census purposes b) Random selection of households in every area survey point
2.	Data base of 36,886 households (Central Statistical Office)—quantity of purchase and consumption of food products in 91 sub-groups (in grams, kilograms, liters) per month in each household
3.	Conversion of consumption into one person per month in each household (in grams, kilograms, liters per person per month)—own calculations
4.	Energy and nutrient content in consumed food products in 91 sub-groups in each household (in kcal, g, mg, µg per day)—own calculations
5.	Average energy and nutrient content in sub-groups in kcal, g, mg, µg per person per day in all households—own calculations
6.	Average energy and nutrient contribution (in %) to the average Polish diet from each sub-group—own calculations
7.	Impact of socio-demographic and economic factors on the level and structure of energy and nutrients supply from milk and dairy products—own calculations

**Table 2:** Main groups and sub-groups in the category of milk and dairy products.

Main Groups	Sub-Groups
Milk	(1) whole milk (2) reduced fat milk (3) condensed and powdered milk
Cheeses and cottage cheeses	(4) ripened and melted cheeses
	(5) cottage cheeses
Yoghurts and milk drinks	(6) yoghurts (7) milk drinks and other dairy products

**Table 3:** Dairy sources of energy contribution to the average Polish diet (in % of total energy contribution).

Contribution of Milk and Dairy Products in %:	Energy
	9.07
**Milk**	**3.21**
whole milk	2.21
reduced fat milk	0.91
condensed and powdered milk	0.09
**Cheeses**	**3.68**
ripened and melted cheeses	2.53
cottage cheeses	1.15
**Yoghurts, milk drinks and other dairy products**	**2.17**
yoghurts	0.58
milk drinks and other dairy products	1.59

**Table 4:** Dairy sources of protein and amino acids contribution to the average Polish diet (in g and %).

Specification	Total Protein	Leucine	Isoleucine	Valine	Lysine	Histidine	Threonine	Tryptophan	Phenylalanine	Methionine	Cysteine	Tyrosine	Arginine	Alanine	Aspartic Acid	Glutamic Acid	Glycine	Proline	Serine
**Average daily supply in g**	77.90	6.04	3.78	4.38	5.29	2.27	3.24	0.98	3.45	1.88	1.18	2.76	4.19	3.80	6.82	15.18	3.38	5.43	3.68
**Contribution of milk and dairy in g**	14.07	1.21	0.72	0.91	1.14	0.39	0.57	0.18	0.66	0.35	0.10	0.65	0.48	0.45	0.97	2.88	0.26	1.35	0.78
**Contribution of milk and dairy in %:**	18.06	20.02	19.05	20.84	21.47	17.02	17.55	18.87	19.21	18.36	8.50	23.61	11.34	11.90	14.29	18.95	7.84	24.80	21.07
**Milk**	5.43	6.15	6.28	6.52	7.42	5.02	5.19	5.37	5.66	5.58	2.81	6.16	3.40	3.84	4.49	5.31	2.27	7.23	6.35
whole milk	3.41	3.87	3.93	4.10	4.47	3.15	3.28	3.39	3.53	3.49	1.76	3.92	2.13	2.44	2.75	3.29	1.36	4.56	4.07
reduced fat milk	1.87	2.12	2.18	2.24	2.74	1.73	1.76	1.83	1.98	1.94	0.98	2.08	1.19	1.30	1.61	1.88	0.84	2.48	2.12
condensed and powdered milk	0.15	0.16	0.17	0.17	0.21	0.14	0.14	0.14	0.15	0.15	0.08	0.16	0.09	0.10	0.13	0.15	0.07	0.19	0.16
**Cheeses**	8.98	9.62	8.74	10.08	9.77	8.77	8.56	9.82	9.66	9.02	3.58	12.97	5.58	5.61	6.75	9.88	3.87	12.84	10.65
ripened and melted cheeses	5.64	5.96	5.35	6.29	6.44	5.76	4.94	6.24	6.23	5.60	2.14	8.33	3.51	3.42	4.18	6.20	2.41	8.18	6.50
cottage cheeses	3.34	3.67	3.39	3.79	3.33	3.00	3.62	3.58	3.43	3.42	1.44	4.64	2.07	2.19	2.57	3.68	1.46	4.65	4.16
**Yoghurts, milk drinks and other dairy products**	3.65	4.24	4.03	4.25	4.28	3.23	3.81	3.69	3.89	3.76	2.12	4.49	2.36	2.46	3.05	3.76	1.70	4.74	4.07
yoghurts	1.05	1.20	1.11	1.23	1.28	0.94	1.07	1.08	1.08	1.07	0.63	1.30	0.64	0.72	0.92	1.09	0.48	1.38	1.19
milk drinks and other dairy products	2.60	3.04	2.92	3.02	3.00	2.28	2.74	2.61	2.81	2.69	1.49	3.19	1.72	1.74	2.14	2.68	1.22	3.36	2.88

**Table 5:** Dairy sources of macronutrients contribution to the average Polish diet (in g or mg and %).

Specification	Carbohydrates	Total Fat	SFA	MUFA	PUFA	Cholesterol
**Average daily supply in g or mg**	270.37 g	96.91 g	34.79 g	37.40 g	17.91 g	316.02 mg
**Contribution of milk and dairy products in g or mg**	13.60 g	10.90 g	6.39 g	3.28 g	0.37 g	36.72 mg
**Contribution of milk and dairy products in %:**	5.03	11.25	18.36	8.77	2.05	11.62
**Milk**	2.28	3.75	6.03	2.85	0.69	4.09
whole milk	1.37	2.96	4.79	2.34	0.57	3.24
reduced fat milk	0.82	0.72	1.13	0.46	0.11	0.75
condensed and powdered milk	0.09	0.07	0.11	0.06	0.01	0.10
**Cheeses**	0.36	5.99	9.90	4.66	1.04	5.69
ripened and melted cheeses	0.02	4.59	7.51	3.61	0.81	4.25
cottage cheeses	0.34	1.40	2.39	1.05	0.23	1.44
**Yoghurts, milk drinks and other dairy products**	2.39	1.50	2.44	1.25	0.32	1.84
yoghurts	0.63	0.39	0.62	0.33	0.10	0.51
milk drinks and other dairy products	1.76	1.12	1.82	0.93	0.22	1.32

**Table 6:** Dairy sources of minerals contribution to the average Polish diet (in mg or µg and %).

Specification	Calcium	Phosphorus	Sodium	Potassium	Magnesium	Iron	Zinc	Copper	Iodine
**Average daily supply in mg or µg**	644.10 mg	1160.19 mg	3,863.84 mg	2,617.85 mg	267.33 mg	10.28 mg	9.78 mg	1.11 mg	154.58 µg
**Contribution of milk and dairy in mg or µg**	352.26 mg	285.64 mg	280.51 mg	310.74 mg	29.35 mg	0.35 mg	1.36 mg	0.06 mg	14.02 µg
**Contribution of milk and dairy products in %**	54.69	24.62	7.26	11.87	10.98	3.44	13.87	5.75	9.07
**Milk**	21.56	9.40	1.44	6.81	5.58	1.23	4.38	2.55	3.01
whole milk	13.70	5.88	0.89	4.11	3.57	0.76	2.58	1.98	1.94
reduced fat milk	7.24	3.25	0.50	2.47	1.84	0.43	1.67	0.50	0.93
condensed and powdered milk	0.62	0.27	0.04	0.23	0.17	0.04	0.13	0.07	0.14
**Cheeses**	21.42	10.09	4.96	1.21	2.51	1.33	6.67	1.67	3.80
ripened and melted cheeses	19.28	7.41	4.37	0.57	1.98	1.02	5.30	1.30	3.32
cottage cheeses	2.14	2.68	0.59	0.64	0.53	0.31	1.37	0.37	0.48
**Yoghurts, milk drinks and other dairy products**	11.72	5.14	0.86	3.85	2.89	0.89	2.81	1,53	2.27
yoghurts	4.37	1.84	0.29	1.55	1.20	0.33	0.89	0.69	0.46
milk drinks and other dairy products	7.35	3.30	0.57	2.30	1.69	0.56	1.92	0.85	1.80

**Table 7:** Dairy sources of vitamin contribution to the average Polish diet (in mg or µg and in%).

Specification	Thiamin	Riboflavin	Niacin	Vitamin B6	Folate	Vitamin B12	Vitamin C	Vitamin A	Vitamin D	Vitamin E
**Average daily supply in mg or µg**	1.32 mg	1.59 mg	16.21 mg	1.84 mg	275.02 µg	4.51 µg	91.40 mg	1,194.55 µg	4.61 µg	13.45 mg
**Contribution of milk and dairy in mg or µg**	0.09 mg	0.45 mg	0.41 mg	0.13 mg	18.07 µg	1.17 µg	2.91 mg	131.28 µg	0.19 µg	0.35 mg
**Contribution of milk and dairy products in %:**	6.67	28.15	2.53	7.12	6.57	26.14	3.18	10.99	4.22	2.61
**Milk**	4.02	13.45	1.53	3.85	2.35	10.28	2.27	4.38	2.11	1.29
whole milk	2.26	8.29	0.69	2.02	1.06	6.38	1.30	3.05	0.95	0.75
reduced fat milk	1.67	4.82	0.81	1.74	1.23	3.69	0.91	1.27	1.15	0.53
condensed and powdered milk	0.10	0.34	0.03	0.09	0.06	0.21	0.07	0.05	0.02	0.01
**Cheeses**	0.73	7.60	0.41	1.30	2.65	9.50	0.03	5.18	1.76	0.96
ripened and melted cheeses	0.38	3.79	0.20	0.58	1.29	6.66	0.00	4.13	1.23	0.68
cottage cheeses	0.35	3.81	0.21	0.72	1.36	2.84	0.03	1.05	0.53	0.28
**Yoghurts, milk drinks and other dairy products**	1.92	7.09	0.58	1.97	1.58	6.35	0.89	1.43	0.35	0.37
yoghurts	0.73	2.51	0.23	0.76	0.58	2.13	0.70	0.48	0.11	0.18
milk drinks and other dairy products	1.18	4.59	0.36	1.22	1.00	4.22	0.19	0.95	0.24	0.19

**Table 8:** Cluster analysis: impact of socio-demographic and economic factors on energy and nutrients contribution from dairy products in the average Polish diet.

Factors	Cramer Correlations
size of the village	0.150
socio-economic affiliation	0.149
land use	0.146
education	0.146
family life phase	0.140
degree of urbanization	0.139
age	0.129
region	0.116
income (quintile group)	0.113
assessment of financial situation	0.099
assessment of nutrition	0.077
number of people in household	0.075
study month	0.053
sex	0.010

**Table 9:** Cluster analysis: consumption of main dairy product groups.

**Specification**	**Sample Population**	**Cluster 1**	**Cluster 2**	**Cluster 3**
Number of households	36,886	7,425	15,804	13,657
Number of persons in households	99,230	20,887	41,321	37,022
**Consumption Per 1 Person Per Month**				
milk (in liters)	3.51	2.73	2.08	5.62
cheeses (in kg)	0.97	1.04	1.15	0.71
yoghurts, milk drinks and other dairy products (in kg)	1.92	4.54	1.12	1.22
